# Obesity and gut microbiome: review of potential role of probiotics

**DOI:** 10.1097/j.pbj.0000000000000111

**Published:** 2021-01-18

**Authors:** Francine Schütz, Margarida Figueiredo-Braga, Pedro Barata, Natália Cruz-Martins

**Affiliations:** a Department of Biomedicine; b Department of Neurosciences and Mental Health, Faculty of Medicine, University of Porto; c Department of Psychiatry, Centro Hospitalar e Universitário de São João; d Fernando Pessoa University; e Institute for Research and Innovation in Health (i3S); f Laboratory of Neuropsychophysiology, Faculty of Psychology and Education Sciences, University of Porto, Porto, Portugal.

**Keywords:** gut microbiota, health promotion, inflammation, obesity, probiotics

## Abstract

Obesity prevalence has increased worldwide over the years, with pandemic levels being already reached, besides to its huge economic and health impacts. The multifactorial pathogenesis of obesity partly explains the important challenge posed to health policy regarding its clinical treatment, with increasing evidences have shown that obesity and metabolic disturbances are closely linked to variations in gut microbiota (GM) function and composition. Indeed, GM play a key contribution in energy metabolism, with GM modulation being increasingly linked to changes in body weight and body mass index. In such matter, probiotics have been proposed as a promising new therapeutic strategy to treat/prevent obesity. Thus, this review aims to provide an overview on the clinical impact and effectiveness of probiotics in obese individuals.

## Introduction

Obesity is a complex, multifactorial pathogenesis, where socioeconomic, hormonal and neuronal mechanisms, unhealthy lifestyle and genetic and epigenetic factors are involved.^1^ According to the World Health Organization, it is estimated that in 2035, 39% of the worldwide adult population will become obese.^2^

Evidences have proposed that obesity and associated metabolic disturbances are correlated to alterations in both gut microbiota (GM) function and composition, which have a crucial role in modulating the host's energy metabolism.^3,4^ Indeed, recently published data have underlined the key determinant role of GM in host's metabolic homeostasis and diseases’ onset.^5^ Indeed, it is a matter of fact that, to GM, a plethora of biological effects have been listed, such as impacting human physiology and pathology; influencing gut epithelial homeostasis; developing host's immunity; modulating host's nutritional status and energy uptake through, for example, producing vitamins and fermenting non-digestible dietary sources; protecting against pathogens; and even promoting drug metabolism.^6–8^ In addition, and namely to what concerns to the use of probiotics, they have emerged as a promising and safe therapy in for different purposes in clinical practice.^7,8^ However, such effectiveness depends on the species and doses used, as well as on underlying disease, with duration of administration also impacting the final outcomes, these ones also varying according to the clinical indication.^7,8^

**Figure 1:**
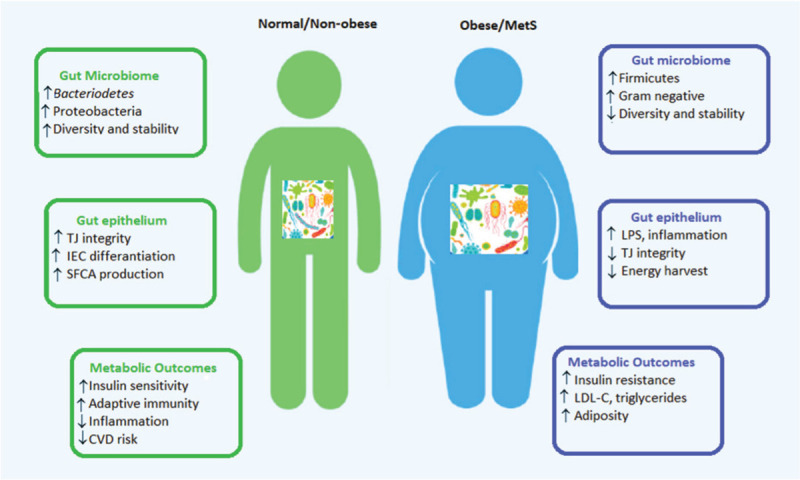
Gut microbiome and the onset of obesity and metabolic syndrome. CVD = cardiovascular disease; IEC = intestinal epithelial cells; LDL-C = low density lipoproteins-cholesterol; LPS = lipopolysaccharide; SCFA = short chain fatty acid; TJ = tight junction.

Nonetheless, the way through whom GM is linked to obesity and metabolic syndrome, and the role of probiotics on both obesity and related consequences prevention and treatment is not yet fully elucidated.^9^ In this sense, this review aims to provide an overview on the clinical impact of probiotics in obese individuals (Figure 1).

## Research methodology

To accomplish with the objective proposed a careful revision of the literature data published in the PubMed database, using “obesity”, “gut microbiota” and “probiotics” as keywords was done. Only clinical (randomized, cohort, case-control) studies published in English in the last 10 years, and addressing the role of probiotics in both obesity and/or metabolic syndrome prevention and treatment were included.

## Gut microbiota and obesity

Briefly, obesity derives from the positive imbalance between energy intake and expenditure leading to a chronic metabolic disease linked to low-grade inflammation. Anyway, the raise in the prevalence of obesity has been mostly linked to socioeconomic factors, sedentary lifestyle and unhealthy dietary habits.^9^ However, recent knowledge has shown an increasing involvement of GM in promoting obesity. Indeed, GM is directly involved in ensuring a proper host's immune function, metabolic activity and appears to also have a key role in the onset of some disorders.^10^

Given that GM has a key role in the normal functioning of immune system and metabolic function, a GM imbalance (dysbiosis) may be viewed as a triggering factor for both metabolic and autoimmune diseases.^11^ In fact, numerous recent studies have clarified the association between body weight (BW) maintenance and GM, and demonstrated that gut microbiome changes decrease BW and fat mass and ameliorate insulin sensitivity.^12^

In 2006, Turnbaugh et al, using a genetic model of obesity, revealed that when germ-free mice were colonized with either the microbiota from genetically obese (ob/ob) mice presented an elevated fat mass and BW.^13^ In fact, perturbations in gut microbiome composition in obese individuals have favored an increased energy harvest from food, and development of adiposity.^14^

Among the mechanisms capable of improving the host's ability to extract energy from the diet is related to the microbiota's ability to break down non-digestible dietary sources, generating short-chain fatty acids (SCFAs) and gases.^15^ As a consequence, SCFAs are able to promote a greater secretion of GLP-1 in the intestine, stimulating the release of insulin from the pancreas, delaying gastric emptying and, thus, promoting satiety and weight loss.^16^ In addition, butyrate, a type of SCFA, as the main source of energy for enterocytes, is also involved in the regulation of cell differentiation and proliferation and promotes the production of GLP-2, reducing intestinal permeability and increasing intestinal glucose transport,^14^ ultimately reducing inflammation and oxidative damage.^17^

In 2005, Ley et al demonstrated that obesity alters GM composition. Comparing the GM composition in genetically obese mice to lean counterparts, the authors stated a reduction in 50% in *Bacteroidetes* and a proportional increase in *Firmicutes* abundance.^18^ In addition, an increase in the number of Gram-negative species in GM has also been reported as a consequence of high-fat diets, promoting greater intestinal absorption of lipopolysaccharides, the increase of which is defined as “metabolic endotoxemia”. Indeed, some studies in animal models have demonstrated that endotoxemia leads to fasted hyperglycemia and hyperinsulinemia, BW gain, like to that found in high-fat-fed mice.^19^

On the other hand, metabolic endotoxemia has been correlated with a low level of intestinal inflammation, due to the interaction between host's immune system and luminal bacteria.^20^ In fact, there have been studies highlighting the important role of inflammation in the main pathophysiological factors that lead to insulin resistance and, consequently, type 2 diabetes.^20,21^ In addition, high-fat diets have been shown to promote the growth of patobionts, triggering an innate immune mediated inflammatory response, that culminate with pro-inflammatory cytokines^21^ or toxic compounds^22^ production.

In addition to the effects described above, these mechanisms also enhance intestinal permeability, microbial molecules translocation and systemic inflammation. Likewise, saturated fatty acids, highly frequent in obesogenic diets, also promote the intestinal permeability by inducing innate immune receptors expression and activation and lymphocytes flow and proliferation.^21^

In humans, several studies have also demonstrated the relationship between variations in GM composition and obesity, where specific alterations in GM structure lead to a proportional increase of *Firmicutes* and to a decrease in *Bacteroidetes* phylum.^23,24^ Also, other studies have shown that subjects with low bacterial richness have higher C-reactive protein and leptin levels, dyslipidemia, insulin resistance, gain more weight and have higher adiposity and inflammatory phenotype comparing to that with high bacterial gene counts.^25^

In general, data have revealed that obese individuals have a GM featured by a decrease in anti-inflammatory bacteria and a raise in pathogens. Such changes trigger a pronounced decrease in SCFAs production, which ultimately impair intestinal barrier integrity and increase mucus degradation potential.^25^

## Probiotic as therapeutic target on obesity

The impact of probiotics on health promotion has been increasingly evident, at the same time that the number of studies focusing on their therapeutic potential in certain diseases has also increased.^26^ In fact, its potential to stimulate the immune system has been highly appreciated, as well as its ability to exert a competitive effect against certain pathogens in addition to many other beneficial effects for the host.^26^ With regard specifically to the probiotics impact and effectiveness on obesity, an increasing number of clinical studies have been done in obese individuals with several associated comorbidities, as described in Table 1.

**Table 1:** Summary of anti-obesity effects of probiotics reported in randomized controlled clinical trials

Population studied	Intervention	Control/placebo group	Duration	Clinical findings	Reference
Adults (33–63 yr): obese tendencies (n = 87); intervention (n = 43); controls (n = 44)	Fermented milk containing *L gasseri* SBT2055	Fermented milk (200 g/d) without probiotic	12 wk	↓ Abdominal visceral, subcutaneous fat areas, BW and BMI	Kadooka et al^27^
Obese children (10–13 yr): hypertransaminasemia and ultrasonographic bright liver (n = 20); intervention (n = 10); placebo (n = 10)	*L rhamnosus* GG (12 billion CFU/d)	1 capsule/d of placebo	8 wk	↓ Hypertransaminasemia, BMI and visceral fat	Vajro et al^37^
Obese adults (40–60 yr): omega-3 fatty acid (n = 15); probiotic VSL#3, (n = 15); omega-3 + probiotic (n = 15); placebo (n = 15)	VSL#3 (*B longum, B infantis, B breve, L acidophilus, L paracasei, L delbrueckii, L plantarum, S salivarius*)	1 capsule/d of placebo	6 wk	↓ TC, TG, LDL, VLDL, and hsCRP ↑ HDL, insulin sensitivity, gut microbiota diversity	Rajkumar et al^31^
Obese adults (20–65 yr); intervention (n = 60); placebo (n = 60)	*L curvatus* HY7601 + *L plantarum* KY1032 + healthy lifestyle	2 cap/d placebo + healthy lifestyle habits	12 wk	↓ BW, WC and fat	Ahn et al^28^
Pre-obese adults (20–64 yr); intervention (n = 40); placebo (n = 40)	*B breve* B-3 (10^10^ CFU/2 cap/d)	2 cap/d of placebo	12 wk	↓ Body fat mass	Minami et al^34^
Overweight and obese adults	*B animalis* ssp. *lactis* 420 (B420) 10^10^ CFU with/without Litesse^®^ Ultra polydextrose (LU) 12 g/d	12 g of LU/d	6 mo	↓ Fat mass and WC↓ Blood hsCRP and zonulin level	Stenman et al^29^
Overweight adults (20–70 yr); Intervention I (n = 21); Intervention II (n = 21); placebo (n = 20)	Intervention I: Living LP28; Intervention II Heat-killed LP28 (*Pediococcus pentosaceus*)	1 capsule/d of placebo	12 wk	↓ BMI, WC after intervention II	Higashikawa et al^33^
Obese and overweight women (18–50 yr): low-fat yogurt (n = 45); probiotic yogurt (n = 44)	Commercial yogurt (*S thermophilus* and *L bulgaricus)* enriched with *L acidophilus* LA5 and *B lactis* BB12	200 g low-fat yogurt/d	12 wk	↓ TC, LDL, insulin resistance	Madjd et al^41^
Obese pregnant women: intervention (n = 25); placebo (n = 25)	Vivomixx^®^ (*S thermophilus, B breve, B longum, B infantis, L acidophilus, L plantarum, L paracasei, L delbrueckii*)	1 capsule/d of placebo	From gestation (14–20 wk) until delivery	↓ Weight gain and pregnancy consequences	Halkjaer et al^46^
Obese women (20–59 yr): intervention (n = 21); placebo (n = 22)	*L acidophilus* LA-14, *L casei* LC-11, *L lactis* LL-23, *B bifidum* BB-06, *B lactis* BL-4 + dietary intervention	1 cap/d placebo + dietary prescription	8 wk	↓ WC	Gomes et al^43^
Obese adults (18–55 yr): intervention (n = 62); placebo (n = 63)	Symbiotic formula (*L rhamnosus,* inulin, oligofructose)	250 mg of maltodextrin + 3 mg magnesium stearate + healthy eating behavior	12 wk	↓ Weight	Sánchez et al^30^
Obese children: intervention (n = 32); placebo (n = 33)	*L rhamnosus, L acidophilus, B bifidum, B lactis*	1 capsule/d of placebo	12 wk	↓ AsAT, AAT, TC, LDL, TG, WC	Famouri et al^38^
Pre-obese-obese, normal weight obese, and normal weight lean women (n = 48); intervention (n = 24); placebo (n = 24)	Psychobiotics (*B bifidum, B animalis, S thermophilus, S thermophilus, L plantarum, L delbrueckii, L reuteri, L acidophilus, L lactis*)	1 capsule/d of placebo	8 wk	↓ BMI, Fat mass, psychopathological scores, bacterial overgrowth and BUT GSI scale↑ Free fat mass, meteorism and defecation frequency	De Lorenzo et al^53^
Obese patients: intervention (n = 23); placebo (n = 23)	Familact^*®*^* (L casei, L rhamnosus, L bulgaricus, L acidophilus, B breve, B longum*, and *S thermophilus)* and FOS	1 sachet/d of placebo	4 wk before and 12 wk post-surgery	↑ Vitamin D, inflammatory, lipid and glycemic indices	Karbaschian et al^52^
Abdominally obese adults: Intervention I (n = 42); Intervention II (n = 44); placebo (n = 40)	*B animalis* subsp *lactis* CECT 8145 (Intervention I) or its heat-killed form (Intervention II)	1 capsule/d of placebo	3 mo	↓ BMI, WC and WC/height ratio	Pedret et al^32^
Obese children (10–15 yr): insulin resistance (n = 48); intervention (n = 23); placebo (n = 25)	*B pseudocatenulatum* + dietary orientations	Placebo + dietary recommendation	13 wk	↓ BW	Sanchis-Chordá et al^39^
Pre-obese adults (20–64 yr): intervention (n = 40); placebo (n = 40)	*B breve* B-3	2 cap/d of placebo	12 wk	↓ Body fat mass	Minami et al^35^
Obese postmenopausal women (45–70 yr): low-dose (n = 27), high-dose (n = 27) intervention; placebo (n = 27)	Low and high dose supplement (*B bifidum, B lactis, B lactis, L acidophilus, L brevis, L casei, L salivarius, L lactis, and L lactis*	1 cap/d of placebo	12 wk	Low-dose group (↓ BW, BMI and fat mass)High-dose (↑ lipid metabolism)	Szulińska et^50^

BMI = body mass index; BW = body weight; CFU = colony-forming units; GLP-1 = glucagon-like peptide-1; HDL = high density lipoprotein; hsCRP = high-sensitivity C reactive protein; LDL = low density lipoprotein; OAGB-MGB = one anastomosis gastric bypass-mini gastric bypass; OFA = omega-3 fatty acid; TC = total cholesterol; TG = triglycerides; VLDL = very low-density lipoprotein; WC = waist circumference.

For example, in a 12-week study period performed in 2010, the administration of fermented milk containing *Lactobacillus gasseri* SBT2055 led to a marked reduction in visceral adipose tissue, BW and body mass index (BMI).^27^ A similar scenario was stated following administration of *L curvatus* HY7601 and *L plantarum* KY1032.^28^ In another study, overweight adults receiving *Bifidobacterium animalis* ssp. *lactis* 420 (B420) with or without fiber (Litesse^®^ Ultra polydextrose) for 6 months revealed a good control in body fat mass.^29^ A similar trend was stated following administration of a symbiotic formula containing *L rhamnosus*, inulin and oligofructose, for 12 weeks, with weight loss being also recorded.^30^

Rajkumar et al using a high-dose mixture of probiotic strains of the *Lactobacillus, Bifidobacterium and Streptococcus* genus along with omega-3 fatty acid supplementation stated a marked improvement in GM composition, plasma lipids, insulin sensitivity, and inflammation markers in overweight adults.^31^ Besides that, the group who received the probiotic alone only demonstrated a positive alteration on GM, while omega-3 alone did not experienced any change.^31^ In abdominally obese individuals, the supplementation of *B animalis* subsp *lactis* CECT 8145 (viable or heat-killed cells) for 3 months markedly improved BMI, waist circumference (WC) and WC/height ratio.^32^ Similar findings were stated after treatment with *heat-killed Pediococcus pentosaceus* LP28 in overweight subjects. In such study, after 12 weeks, the participants showed a reduction in BMI, body fat and WC.^33^

Positive outcomes in decreasing body fat mass were also stated following treatment with *Bifidobacteriu breve* B-3 in pre-obese adults after 12 weeks. Concomitantly, improvements in inflammation- and liver function-related parameters were also listed.^34^ Such results were corroborated in another study in healthy pre-obese individuals where *B breve* B-3 led to body fat reduction.^35^ In such way, Kondo and colleagues, in a intend to describe the possible anti-obesity mechanisms of *B breve* B-3 underlined that they were triggered by intestinal barrier function improvement, B-3-derived metabolites (acetic and conjugated linoleic acids), and adiponectin and pro-glucagon production.^36^

In hepatopathic obese children, the supplementation with high-dose *L rhamnosus* GG along with lifestyle intervention for 8 weeks led to a marked reduction in hypertransaminasemia.^37^ Similarly, another study in obese children with nonalcoholic fatty liver disease (NAFLD) stated that the administration of a probiotic mixture composed of *L rhamnosus* DSMZ 21690, *L acidophilus* ATCCB3208, *B bifidum* ATCC SD6576 and *B lactis* DSMZ 32269 for 12 weeks decreased aspartate transaminase, alanine aminotransferase, triglycerides, total cholesterol, and LDL-C levels, and WC.^38^ Other authors assessing the effect of *B pseudocatenulatum* CECT 7765 in insulin-resistant obese children over a 13-week period and reported a pronounced reduction in BW and improvement in inflammatory status.^39^

Besides to the above-mentioned findings, probiotics have also revealed great abilities to reverse dysbiosis and normalize GM, which explain for example the protective effects reported in NAFLD patients (with endotoxemia and inflammation markers reduction).^40^ In addition, some probiotics have shown a great potential to reduce hepatic fat deposition, also exerting anti-fibrotic effects by modulating collagen expression and transforming growth factor-beta.^40^

Another study carried out in obese and overweight women reported that the consumption of a yogurt commercially available (*Streptococcus thermophilus and L bulgaricus*) enriched with *L acidophilus* LA5 and *B lactis* BB12 for 12 weeks improved both lipid profile and insulin sensitivity.^41^ Indeed, there are increasing evidences reporting that probiotics can modify bile acid metabolism, which ultimately influences cholesterol absorption in the gut lumen. The hypocholesterolemic effect of probiotics can be explained by the fact that some bacterial species produced an enzyme, bile salt hydrolase, involved in the first reaction biliary salts deconjugation.^42^

In obese women, the administration of a probiotic mix composed of *L acidophilus LA-14, L casei LC-11, L lactis LL-23,* and *B bifidum* BB*-*06, improved BW composition after an 8-week supplementation period. As main findings, the study group revealed a marked decrease in WC, WC*-*height ratio and conicity index, due to suppression of fasting*-*induced adipose factor in the gut, involved in SCFAs production.^43^ In fact, evidence has shown that SCFAs regulate energy metabolism by interaction with G protein*-*coupled receptors, thus modifying insulin sensitivity both in adipocytes and peripheral organs.^44^^.^^45^

In obese pregnant women, Vivomixx^®^ (a commercial product with 8 probiotic bacterial strains) promoted a control in weight gain. Besides that, the probiotic mix could reduce complications during pregnancy.^46^ Other study demonstrated that the daily intake of probiotics could impair the preeclampsia risk in primiparous women.^47^ Similarly, Asemi et al, demonstrated that the daily intake of a probiotic yoghurt promoted serum insulin levels maintenance and helped pregnant women from insulin resistance.^48^ Some other probiotic strains have also revealed great abilities to decrease glucose absorption and to improve insulin resistance through direct action on upregulated proglucagons expression, decreasing adiposity and inflammation status.^49^

In obese postmenopausal women, the consumption of a probiotic mix Ecologic^®^, composed of 9 *Lactobacillus* and *Bifidobacterium* strains for 12 weeks ameliorated BW, BMI, visceral fat, and fat mass, although the most prominent changes were stated to the group receiving the high-dose probiotic.^50^ Interestingly, such changes in BW composition can be explained by the probiotic mix ability to decrease SCFAs accumulation through GPR43 interaction, resulting in an increased adipogenesis and leptin release and decreased lipolysis and inflammation.^51^ Furthermore, the SCFAs interaction with G protein-coupled receptor lead to an enhanced satiety by raising the intestinal secretion of glucagon-like peptide 1 and polypeptide YY.^51^

In morbid obese patients following gastric bypass surgery a probiotic supplement Familact^®^ promoted BW loss and ameliorated pro-inflammatory biomarker (TNF-α) and vitamin D status.^52^ The possible mechanisms behind such effects appeared to be due to direct impact on gastrointestinal appetite hormones and gut microbiome composition, which ultimately modify both diet-harvested energy and promote host’ energy homeostasis.^51^

De Lorenzo et al, in a 3-week supplementation period with a mixture including 10 different psychobiotics revealed a prominent modulation in BW composition. As main findings, psychobiotics led to a pronounced reduction in BMI, resistance and fat mass, and to an increase in free fat mass, along with a decrease in bacterial overgrowth and psychopathological scores.^53^

Also worth of note is that the microbiota-gut-brain axis, while a bidirectional communication pathway between GM and brain,^54^ have been target of a high attention. Indeed, increasing evidence have shown that GM exerts a crucial role in both mood and behavior regulation, besides GM modulation.^55,56^ For example, Di Renzo et al, found an association between BW composition and eating behavior in normal weight obese patients, which appears to be related to their ability to mitigate anxiety symptoms and improve eating behavior, ultimately contributing to weight loss.^57^

In short, taking into account the currently available clinical data, probiotic supplementation seems to be useful to improve specific anthropometric, biochemical and inflammatory parameters, although the absence of some consistent data, of detailed information related to the different probiotic strains used, and lack of long-term follow-up and high sample size studies limit the establishment of more solid conclusions.

## Conclusions

Intestinal microbiota has a preponderant role in both modulating fat deposition and energy homeostasis, as well as in the host's relationship with environmental factors. Recent data have revealed that thin individuals have an intestinal microbiota composition completely different from that observed in obese individuals, which directly relate dysbiosis to the greater propensity to obesity and linked metabolic disorders. In addition, more and more studies are showing the beneficial effects of the consumption of probiotics in maintaining BMI, BW, fat mass, WC, lipid profile and inflammatory status. Taken together, these data point to new horizons regarding intervention on obesity and related metabolic disorders, despite more studies are needed to assertively assess the effect of a plethora of probiotic strains or symbiotic preparations for both health promotion and treatment.

## Acknowledgements

N.M. acknowledges the Portuguese Foundation for Science and Technology under the Horizon 2020 Program (PTDC/PSI-GER/28076/2017).

## Conflicts of interest

Authors have declared no conflict of interest.

## References

[1] Cerdó T García-Santos JA GBermúdez M Campoy C The role of probiotics and prebiotics in the prevention and treatment of obesity. Nutrients. 2019;11:635. 10.3390/nu11030635 PMC6470608 30875987

[2] Obesity: preventing and managing the global epidemic. Report of a WHO consultation. World Health Organ Tech Rep Ser. 2000;894: i–xii; 1–253. 11234459

[3] Festi D Schiumerini R Eusebi LH Marasco G Taddia M Colecchia A Gut microbiota and metabolic syndrome. World J Gastroenterol. 2014;20:16079–16094. 25473159 10.3748/wjg.v20.i43.16079 PMC4239493

[4] Saklayen MG The global epidemic of the metabolic syndrome. Curr Hypertens Rep. 2018;20:12. 29480368 10.1007/s11906-018-0812-z PMC5866840

[5] Cani PD Delzenne NM Gut microflora as a target for energy and metabolic homeostasis. Curr Opin Clin Nutr Metab Care. 2007;10:729–734. 18089955 10.1097/MCO.0b013e3282efdebb

[6] Sanz Y Olivares M Moya-Pérez Á Agostoni C Understanding the role of gut microbiome in metabolic disease risk. Pediatr Res. 2015;77:236–244. 25314581 10.1038/pr.2014.170

[7] Salehi B Dimitrijević M Aleksić A Human microbiome and homeostasis: insights into the key role of prebiotics, probiotics, and symbiotics. Crit Rev Food Sci Nutr. 2020;13:1–14. 10.1080/10408398.2020.1760202 32400169

[8] Sharifi-Rad J Rodrigues CF Stojanović-Radić Z Probiotics: versatile bioactive components in promoting human health. Medicina (Kaunas). 2020;56:E433. 32867260 10.3390/medicina56090433 PMC7560221

[9] Green M Arora K Prakash S Microbial medicine: prebiotic and probiotic functional foods to target obesity and metabolic syndrome. Int J Mol Sci. 2020;21:2890. 10.3390/ijms21082890 PMC7215979 32326175

[10] Ellulu MS Patimah I Khaza’ai H Rahmat A Abed Y Obesity and inflammation: the linking mechanism and the complications. Arch Med Sci. 2017;13:851–863. 28721154 10.5114/aoms.2016.58928 PMC5507106

[11] Abenavoli L, Scarpellini E, Colica C, Boccuto L, Salehi B, Sharifi-Rad J, Aiello V, Romano B, De Lorenzo A, Izzo AA, Capasso R. Gut microbiota and obesity: a role for probiotics. Nutrients. 2019; 7:2690. 10.3390/nu11112690 PMC6893459 31703257

[12] Rastelli M Knauf C Cani PD Gut microbes and health: a focus on the mechanisms linking microbes, obesity, and related disorders. Obesity (Silver Spring). 2018;26:792–800. 29687645 10.1002/oby.22175 PMC5947576

[13] Turnbaugh PJ Ley RE Mahowald MA Magrini V Mardis ER Gordon JI An obesity-associated gut microbiome with increased capacity for energy harvest. Nature. 2006;444:1027–1031. 17183312 10.1038/nature05414

[14] Turnbaugh PJ Bäckhed F Fulton L Gordon JI Diet-induced obesity is linked to marked but reversible alterations in the mouse distal gut microbiome. Cell Host Microbe. 2008;3:213–223. 18407065 10.1016/j.chom.2008.02.015 PMC3687783

[15] Larraufie P Martin-Gallausiaux C Lapaque N SCFAs strongly stimulate PYY production in human enteroendocrine cells. Sci Rep. 2018;8:74. 29311617 10.1038/s41598-017-18259-0 PMC5758799

[16] Cani PD Possemiers S Van de Wiele T Changes in gut microbiota control inflammation in obese mice through a mechanism involving GLP-2-driven improvement of gut permeability. Gut. 2009;58:1091–1103. 19240062 10.1136/gut.2008.165886 PMC2702831

[17] Singh RK Chang HW Yan D Influence of diet on the gut microbiome and implications for human health. J Transl Med. 2017;15:73. 28388917 10.1186/s12967-017-1175-y PMC5385025

[18] Ley RE Bäckhed F Turnbaugh P Lozupone CA Knight RD Gordon JI Obesity alters gut microbial ecology. Proc Natl Acad Sci USA. 2005;102:11070–11075. 16033867 10.1073/pnas.0504978102 PMC1176910

[19] Cani PD Amar J Iglesias MA Metabolic endotoxemia initiates obesity and insulin resistance. Diabetes. 2007;56:1761–1772. 17456850 10.2337/db06-1491

[20] de La Serre CB Ellis CL Lee J Hartman AL Rutledge JC Raybould HE Propensity to high-fat diet-induced obesity in rats is associated with changes in the gut microbiota and gut inflammation. Am J Physiol Gastrointest Liver Physiol. 2010;299:G440–G448. 20508158 10.1152/ajpgi.00098.2010 PMC2928532

[21] Kim KA Gu W Lee IA Joh EH Kim DH High fat diet-induced gut microbiota exacerbates inflammation and obesity in mice via the TLR4 signaling pathway. PLoS One. 2012;7:e47713. 23091640 10.1371/journal.pone.0047713 PMC3473013

[22] Devkota S Wang Y Musch MW Dietary-fat-induced taurocholic acid promotes pathobiont expansion and colitis in Il10–/– mice. Nature. 2012;487:104–108. 22722865 10.1038/nature11225 PMC3393783

[23] Armougom F Henry M Vialettes B Raccah D Raoult D Monitoring bacterial community of human gut microbiota reveals an increase in Lactobacillus in obese patients and Methanogens in anorexic patients. PLoS One. 2009;4:e7125. 19774074 10.1371/journal.pone.0007125 PMC2742902

[24] Santacruz A Collado MC García-Valdés L Gut microbiota composition is associated with body weight, weight gain and biochemical parameters in pregnant women. Br J Nutr. 2010;104:83–92. 20205964 10.1017/S0007114510000176

[25] Le Chatelier E Nielsen T Qin J Richness of human gut microbiome correlates with metabolic markers. Nature. 2013;500:541–546. 23985870 10.1038/nature12506

[26] Hill C Guarner F Reid G Expert consensus document. The International Scientific Association for Probiotics and Prebiotics consensus statement on the scope and appropriate use of the term probiotic. Nat Rev Gastroenterol Hepatol. 2014;11:506–514. 24912386 10.1038/nrgastro.2014.66

[27] Kadooka Y Sato M Imaizumi K Regulation of abdominal adiposity by probiotics (Lactobacillus gasseri SBT2055) in adults with obese tendencies in a randomized controlled trial. Eur J Clin Nutr. 2010;64:636–643. 20216555 10.1038/ejcn.2010.19

[28] Ahn HY Kim M Chae JS Supplementation with two probiotic strains, Lactobacillus curvatus HY7601 and Lactobacillus plantarum KY1032, reduces fasting triglycerides and enhances apolipoprotein A-V levels in non-diabetic subjects with hypertriglyceridemia. Atherosclerosis. 2015;241:649–656. 26117402 10.1016/j.atherosclerosis.2015.06.030

[29] Stenman LK Lehtinen MJ Meland N Probiotic with or without fiber controls body fat mass, associated with serum zonulin, in overweight and obese adults-randomized controlled trial. EBioMedicine. 2016;13:190–200. 27810310 10.1016/j.ebiom.2016.10.036 PMC5264483

[30] Sanchez M Darimont C Panahi S Effects of a diet-based weight-reducing program with probiotic supplementation on satiety efficiency, eating behaviour traits, and psychosocial behaviours in obese individuals. Nutrients. 2017;9:284. 10.3390/nu9030284 PMC5372947 28294985

[31] Rajkumar H Mahmood N Kumar M Varikuti SR Challa HR Myakala SP Effect of probiotic (VSL#3) and omega-3 on lipid profile, insulin sensitivity, inflammatory markers, and gut colonization in overweight adults: a randomized, controlled trial. Mediators Inflamm. 2014;2014:348959. 24795503 10.1155/2014/348959 PMC3984795

[32] Pedret A Valls RM Calderón-Pérez L Effects of daily consumption of the probiotic Bifidobacterium animalis subsp. lactis CECT 8145 on anthropometric adiposity biomarkers in abdominally obese subjects: a randomized controlled trial. Int J Obes (Lond). 2019;43:1863–1868. 30262813 10.1038/s41366-018-0220-0 PMC6760601

[33] Higashikawa F Noda M Awaya T Antiobesity effect of Pediococcus pentosaceus LP28 on overweight subjects: a randomized, double-blind, placebo-controlled clinical trial. Eur J Clin Nutr. 2016;70:582–587. 26956126 10.1038/ejcn.2016.17

[34] Minami J Kondo S Yanagisawa N Oral administration of Bifidobacterium breve B-3 modifies metabolic functions in adults with obese tendencies in a randomised controlled trial. J Nutr Sci. 2015;4:e17. 26090097 10.1017/jns.2015.5 PMC4463018

[35] Minami J Iwabuchi N Tanaka M Effects of Bifidobacterium breve B-3 on body fat reductions in pre-obese adults: a randomized, double-blind, placebo-controlled trial. Biosci Microbiota Food Health. 2018;37:67–75. 30094122 10.12938/bmfh.18-001 PMC6081611

[36] Kondo S Xiao J Satoh T Antiobesity effects of Bifidobacterium breve strain B-3 supplementation in a mouse model with high-fat diet-induced obesity. Biosci Biotechnol Biochem. 2010;74:1656–1661. 20699581 10.1271/bbb.100267

[37] Vajro P Mandato C Licenziati MR Effects of Lactobacillus rhamnosus strain GG in pediatric obesity-related liver disease. J Pediatr Gastroenterol Nutr. 2011;52:740–743. 21505361 10.1097/MPG.0b013e31821f9b85

[38] Famouri F Shariat Z Hashemipour M Keikha M Kelishadi R Effects of probiotics on nonalcoholic fatty liver disease in obese children and adolescents. J Pediatr Gastroenterol Nutr. 2017;64:413–417. 28230607 10.1097/MPG.0000000000001422

[39] Sanchis-Chordà J Del Pulgar EMG Carrasco-Luna J Benítez-Páez A Sanz Y Codoñer-Franch P Bifidobacterium pseudocatenulatum CECT 7765 supplementation improves inflammatory status in insulin-resistant obese children. Eur J Nutr. 2019;58:2789–2800. 30251018 10.1007/s00394-018-1828-5

[40] Xie C Halegoua-DeMarzio D Role of probiotics in non-alcoholic fatty liver disease: does gut microbiota matter? Nutrients. 2019;11:2837. 10.3390/nu11112837 PMC6893593 31752378

[41] Madjd A Taylor MA Mousavi N Comparison of the effect of daily consumption of probiotic compared with low-fat conventional yogurt on weight loss in healthy obese women following an energy-restricted diet: a randomized controlled trial. Am J Clin Nutr. 2016;103:323–329. 26702123 10.3945/ajcn.115.120170

[42] Pavlović N Stankov K Mikov M Probiotics—interactions with bile acids and impact on cholesterol metabolism. Appl Biochem Biotechnol. 2012;168:1880–1895. 23054820 10.1007/s12010-012-9904-4

[43] Gomes AC de Sousa RG Botelho PB Gomes TL Prada PO Mota JF The additional effects of a probiotic mix on abdominal adiposity and antioxidant Status: A double-blind, randomized trial. Obesity (Silver Spring). 2017;25:30–38. 28008750 10.1002/oby.21671

[44] Bäckhed F Ding H Wang T The gut microbiota as an environmental factor that regulates fat storage. Proc Natl Acad Sci USA. 2004;101:15718–15723. 15505215 10.1073/pnas.0407076101 PMC524219

[45] Canfora EE Jocken JW Blaak EE Short-chain fatty acids in control of body weight and insulin sensitivity. Nat Rev Endocrinol. 2015;11:577–591. 26260141 10.1038/nrendo.2015.128

[46] Halkjaer SI Nilas L Carlsen EM Effects of probiotics (Vivomixx® in obese pregnant women and their newborn: study protocol for a randomized controlled trial. Trials. 2016;17:491. 27724923 10.1186/s13063-016-1617-5 PMC5057415

[47] Brantsaeter AL Myhre R Haugen M Intake of probiotic food and risk of preeclampsia in primiparous women: the Norwegian Mother and Child Cohort Study. Am J Epidemiol. 2011;174:807–815. 21821542 10.1093/aje/kwr168 PMC3203379

[48] Asemi Z Samimi M Tabassi Z Effect of daily consumption of probiotic yoghurt on insulin resistance in pregnant women: a randomized controlled trial. Eur J Clin Nutr. 2013;67:71–74. 23187955 10.1038/ejcn.2012.189

[49] Esteve E Ricart W Fernández-Real JM Gut microbiota interactions with obesity, insulin resistance and type 2 diabetes: did gut microbiote co-evolve with insulin resistance? Curr Opin Clin Nutr Metab Care. 2011;14:483–490. 21681087 10.1097/MCO.0b013e328348c06d

[50] Szulińska M Łoniewski I van Hemert S Sobieska M Bogdański P Dose-dependent effects of multispecies probiotic supplementation on the lipopolysaccharide (LPS) level and cardiometabolic profile in obese postmenopausal women: a 12-week randomized clinical trial. Nutrients. 2018;10:773. 10.3390/nu10060773 PMC6024794 29914095

[51] Arora T Singh S Sharma RK Probiotics: Interaction with gut microbiome and antiobesity potential. Nutrition. 2013;29:591–596. 23287068 10.1016/j.nut.2012.07.017

[52] Karbaschian Z Mokhtari Z Pazouki A Probiotic supplementation in morbid obese patients undergoing one anastomosis gastric bypass-mini gastric bypass (OAGB-MGB) surgery: a randomized, double-blind, placebo-controlled. Clinical Trial Obes Surg. 2018;28:2874–2885. 10.1007/s11695-018-3280-2 29725975

[53] De Lorenzo A Costacurta M Merra G Can psychobiotics intake modulate psychological profile and body composition of women affected by normal weight obese syndrome and obesity? A double blind randomized clinical trial. J Transl Med. 2017;15:135. 28601084 10.1186/s12967-017-1236-2 PMC5466767

[54] Colica C Avolio E Bollero P Evidences of a new psychobiotic formulation on body composition and anxiety. Mediators Inflamm. 2017;2017:5650627. 29147070 10.1155/2017/5650627 PMC5632987

[55] Cryan JF Dinan TG Mind-altering microorganisms: the impact of the gut microbiota on brain and behaviour. Nat Rev Neurosci. 2012;13:701–712. 22968153 10.1038/nrn3346

[56] Park AJ Collins J Blennerhassett PA Altered colonic function and microbiota profile in a mouse model of chronic depression. Neurogastroenterol Motil. 2013;25:733-e575. 23773726 10.1111/nmo.12153 PMC3912902

[57] Di Renzo L Tyndall E Gualtieri P Association of body composition and eating behavior in the normal weight obese syndrome. Eat Weight Disord. 2016;21:99–106. 26347355 10.1007/s40519-015-0215-y

